# Bayesian theories of consciousness: a review in search for a minimal unifying model

**DOI:** 10.1093/nc/niab038

**Published:** 2021-10-13

**Authors:** Wiktor Rorot

**Affiliations:** Faculty of Philosophy and Faculty of Psychology, University of Warsaw, ul. Krakowskie Przedmieście 3, 00-927, Stawki 5/7, Warsaw 00-183, Poland

**Keywords:** consciousness, free energy principle, active inference, predictive processing, computational explanation, minimal unifying model

## Abstract

The goal of the paper is to review existing work on consciousness within the frameworks of Predictive Processing, Active Inference, and Free Energy Principle. The emphasis is put on the role played by the precision and complexity of the internal generative model. In the light of those proposals, these two properties appear to be the minimal necessary components for the emergence of conscious experience—a Minimal Unifying Model of consciousness.

## Introduction

[T]his is not mysterious, it is just a mathematical statement of the way things are. (Friston *et al.* 2020, 11)

The goal of this paper is to provide an overview of the research on consciousness—with specific focus on phenomenal consciousness (Block 1995)^1^—that has been so far carried out within the formal schema of the Free Energy Principle (FEP) (e.g. Friston 2009, 2010, 2019).

Over the past 15 years, the FEP—together with related frameworks stemming from the same theoretical core, namely the Predictive Processing (PP; e.g. Hohwy 2013; Clark 2016; Wiese and Metzinger 2017) and Active Inference Framework (e.g. Friston *et al.* 2017a)^2^—has become one of the central approaches in cognitive neuroscience, neurobiology, and philosophy of mind and an increasingly important approach to artificial intelligence, specifically reinforcement learning (see e.g. Ueltzhöffer 2018; Millidge 2019). I will refer to these theories with the name “Bayesian cognitive science,” coined by Ramstead *et al.* (2020), despite the fact that the FEP does not exhaust all possibilities for “Bayesian” theories of cognition and consciousness. Although there is much debate about the general viability and the *de facto* meaning of this framework (see e.g. Colombo and Wright 2018; Litwin and Miłkowski 2020; Andrews 2021), it currently seems that it is here to stay for the foreseeable future.

In a recent paper, Hohwy (2020) discusses the current focus of the FEP, as well as emerging new areas of research within this framework. Among them, he lists the growing body of work on consciousness. This work spans several, mutually exclusive, philosophical perspectives on subjective experience, as well as touches on issues common to many of them, such as the problem of identifying neural correlates of consciousness (Hohwy and Seth 2020). In this work, the FEP exhibits its “attractive duality” (Hohwy 2020, 11)—i.e. its orthogonality to extant lines of argument.

Surprisingly, despite the disparities, all those proposals are based on similar elements of the FEP formalism - in particular, the concepts of “precision” and “complexity” (in the technical sense of those two terms, described in the next section). Wanja Wiese (2020) has advanced the notion of a “minimal unifying model” as a framework for building a consensus between various accounts of consciousness. In this paper, I will review the existing body of work on consciousness in the Bayesian cognitive science. Based on this review, I will argue that these two properties of internal models—precision and complexity—do in fact constitute a minimal unifying model at least for Bayesian cognitive science of consciousness. In this way, this body of work can be interpreted as making coherent (albeit limited), empirically testable predictions about the neural mechanisms of consciousness.

In what follows, in the “A very short introduction to the FEP” section, I will provide a very short introduction to the FEP, focusing on the common elements of the three frameworks of Bayesian cognitive science. Next, in the “Conscious experience under the FEP” section, I will provide an overview of the theories of consciousness that employ these frameworks. In the “Discussion” section, I will discuss the Bayesian theories of consciousness in general, trying to gesture towards a common approach within those different accounts. Finally, in the “Precision and complexity as a Minimal Unifying Model” section, I will discuss the interpretation of the core of this common approach in terms of a minimal unifying model, which I believe to be the most informative.

## A very short introduction to the FEP

The FEP (Friston 2019) states a simple tautology: any living system, as long as it stays alive, can be described as minimizing the dispersion of its constituent states. For the purpose of this definition, “living system” is understood as one that is in a nonequilibrium steady state (NESS), and staying alive means maintaining this status. To do this, the system has to remain far from the thermodynamic equilibrium in a (relatively) low entropy state (hence the tautology). Furthermore, the FEP cashes out the Good Regulator theorem (Conant and Ross Ashby 1970), which states that every good regulator of a system must be isomorphic with that system (i.e. must be a model of that system). As long as it remains far from thermodynamic equilibrium, the system behaves as if it employed or instantiated^3^ a *generative model*, an internal statistical representation of the target NESS density (and the worldly influences). Effectively, the system can be described as capable of anticipating the changes it undergoes and of adaptive behavior. The principle has been applied to a wide range of self-organizing systems (e.g. Friston and Stephan 2007; Friston 2013; Friston *et al.* 2015a; Kirchhoff and Kiverstein 2018; Fields and Levin 2019). However, from the philosophy of science perspective, it is best regarded as a modeling framework rather than a model per se [Litwin and Miłkowski (2020) and Andrews (2021) make a compelling argument in this direction].

With regard to cognition, the “Bayesian cognitive science” paints the picture of the brain as a hierarchical “prediction machine.”^4^ Using its information about the state of the world, specifically about the hidden causes of sensory input—formally represented by probability distributions referred to as the *recognition density* and the *generative model*—the brain (or the embodied cognitive system) attempts to predict the incoming stimuli. The mismatch between prediction and input, the *prediction error*, is then passed from the bottom up along the neural hierarchy and is used to bring the recognition density closer to the actual distribution of causes either via an update of the internal model itself—*perceptual inference*—or through active involvement with the world—*active inference*—which attempts to bring the hidden causes closer to the generative density (one can think of it as a self-fulfilling prophecy). The idea of the interplay between top-down prediction and bottom-up prediction error is at the core of the applications of the framework to actual neural circuits, as is the case with e.g. the predictive coding model of vision (Rao and Ballard 1999).

Prediction errors indicate *surprisal* (in the technical sense of information theory, mathematically equivalent to entropy), and this indicates that the system is leaving the NESS. Hence, the minimization of surprisal follows strictly from the formulation of the FEP. However, since this value is intractable, an approximation must be used. The *(variational) free energy* provides an upper bound on surprisal by measuring the difference between recognition and generative density.

This free energy functional can be formally decomposed into two equivalent formulations (see e.g. Friston 2009), corresponding to the aforementioned perceptual and active inference, which can be symbolically depicted as:

$$\begin{equation*}F = Divergence + Surprise\vspace*{-8pt}\end{equation*}$$

$$\begin{equation*}F = Complexity - Accuracy\vspace*{6pt}\end{equation*}$$

The latter decomposition is crucial for our current purposes. These terms are defined as follows:

*Complexity* denotes the amount of information required to reconstruct the generative density, given a full knowledge of the recognition density (quantified with a Kullback–Leibler divergence between the two densities).

*Accuracy* denotes how close are the predictions generated by the internal model to the actual distribution of probabilities of observations, given the hidden causes.

Thus, the process of active inference can be understood as the process of selectively sampling the world to bring sensations closer to what is expected (note that, throughout the paper, the terms “precision” and “complexity” are used only in the technical sense described here and below.)

However, the increase in accuracy is bound to an increase in complexity and to the risk of overfitting the internal model. This fact comes simply from the irreducible uncertainty and randomness of the world. To take it into account, the model has to give up some accuracy (and complexity) in exchange for robustness.

Before I move to the discussion of how the Bayesian cognitive science tries to explain consciousness, I will provide an example of an explanation of attention employing the FEP (roughly falling under the PP label), showcasing crucial components of this framework and introducing further notions that will turn out central later on.^5^

### Attention under the FEP

PP explains attention in terms of the control of precision assigned to top-down hypotheses and bottom-up prediction error signals (Feldman and Friston 2010; Hohwy 2012, 2013). Formally speaking, *precision* is the inverse variance of a probability distribution.^6^ Precision, most notably describes the reliability of the prediction error signals (sensory noise) and the hypothesis generated from the generative density (neural noise). Despite both cases being formally defined in terms of inverse variance, precision assignments are independent and, as such, have to be independently tracked by the cognitive system.^7^ In the more complex case of active inference, Parr and Friston (2017a,b) identify three types of precision assignments, most importantly sensory and expected free energy precision (see also Smith *et al.* 2021),^8^ linked to different neurotransmitter systems in the brain and responsible for mechanisms of attention (Parr and Friston 2017b).

Since precisions are not given and need to be inferred from the available data, they are also subject to control from the system itself—especially since they are volatile between context and sensory modalities. As we learn from the Good Regulator theorem, if the brain is supposed to manage precision expectations (on a sub-personal level), then they have to be somehow modeled—either directly, in the form of precision expectations (as Hohwy 2012 argues), or indirectly, in the form of the hyperparameters of the generative density (Buckley *et al.* 2017). This process can also be regarded as somewhat disjointed and separate and hence interpreted as a specialized model for managing precisions [this point is leveraged by Dołęga and Dewhurst (2019) as we will see below].

Under this description, exogenous attention—the experience of a stimulus “with a large spatial and/or temporal contrast (abrupt onset)” (Hohwy 2012, 6)—arises due to the expectation that stronger signals should have a larger signal to noise ratio, and hence a better (sensory) precision. On the other hand, endogenous attention—the top-down, internally governed assignment of focus—operates by directly modulating the responses of neuronal units encoding beliefs about (expected free energy) precision (Hohwy 2012, 7), effectively assigning higher precision to the relevant spatiotemporal regions.

Hohwy (2012) discusses several examples of inattentional blindness to show in detail how this account of attention can explain them. Here, I will briefly recount his account of change blindness to make the somewhat formal discussion above more down to earth.

Consider an experiment where the participant is presented two similar images of an airplane. The second image differs in that it is covered in mudsplashes, as well as the airplane depicted misses an engine. Since the distractor—mudsplashes—offers a strong signal and is a relatively more visible (larger or more salient) change than the missing engine, it will “grab” the attention, i.e. high precision will be assigned to the corresponding parts of the signal. In result, this change will activate a specific hypothesis about the world—that the participant is presented with a transient occlusion or a change to the photo, with a strong prior belief that the photo beneath the mudsplashes remains the same. Only after this hypothesis is accepted as the model, and the prediction errors corresponding to unexpected appearance of mudsplashes are explained away, the subtle change corresponding to the absence of the engine can be assigned a higher precision and thus—can be brought to the participant’s attention.

## Conscious experience under the FEP

There are several competing models that attempt to account for conscious experience—specifically for the phenomenal consciousness—employing tools provided by the Bayesian cognitive science. These are:

1. Bayesian implementation of Graziano’s Attention Schema Theory (B-AST) (Graziano 2013; Dołęga and Dewhurst 2019, 2021);
2. higher-order order state space approach (HOSS) (Fleming 2020);
3. the “winning hypothesis” (WH) account (Hohwy 2012, 2013);
4. Predictive Global Neuronal Workspace theory (PGNW) (Hohwy 2013, 2015; Whyte 2019; Whyte and Smith 2021);
5. the “generative entanglement” (GE) account (Clark 2018, 2019; Clark *et al.* 2019);
6. Projective Consciousness Model (PCM) (Rudrauf *et al.* 2017, 2020; Williford *et al.* 2018);
7. Integrated World Modeling Theory (IWMT) (Safron 2020);
8. Dual Aspect Monism (DAM) (Solms and Friston 2018; Solms 2019; see also Chanes and Barrett 2016);
9. Markovian Monism (MM) (Friston *et al.* 2020).^9^

The list above is organized roughly along the axis of the degree of realism of the accounts—as I have previously mentioned, the FEP research on consciousness is characterized by “attractive duality” in that it is orthogonal to extant distinctions.^10^ Space considerations do not allow for a full discussion of all the accounts listed above. I will, however, attempt to provide the gist of each.

It is important to note that these accounts address different aspects of phenomenal consciousness, so they are not entirely competing with one another. However, many of them overlap in some respects providing incoherent solutions to similar problems.

Finally, an important *caveat*: many of the theories of consciousness discussed below follow a realist interpretation of the FEP, arguing that brains (or embodied cognitive systems) actually do perform free energy (FE) minimization—e.g. Williford *et al.* (2018) uses the claim that “some quantity is being optimized” to argue for realism about the computational theory of mind. In some cases, this is justified by the authors’ reliance on specific process theories of FEP (PP and Active Inference Framework; see Andrews 2021); however, in general, such an approach does not stand up to scrutiny [as Andrews (2021) and van Es (2020) convincingly argue].

### Bayesian Attention Schema Theory (AST)

AST has been originally proposed by Graziano (2013). Under this view, consciousness is subjective awareness, and it results from the subject attending to a given stimulus. More precisely, access consciousness is realized by attention, while the structure of first-person experience with associated phenomenology is explained by the properties of the process of control of attention. Control of attention falls upon an internal model called the “attention schema,” conceived of in analogy to the internal model of the body, namely—the body schema. This means that the subject has access to a simplified model of how attention is deployed, which it can look into whenever a report of what is it conscious of is required. And, according to AST, whenever we claim to be conscious of something, we are in fact using higher-order cognition to “introspect” our attention schema and report what information it holds. This proposal fits well with PP explanation of attention, described above, and, hence, a Bayesian implementation of AST has been recently proposed (Dołęga and Dewhurst 2019, 2021).

A crucial part of both original AST and its PP formalization is the account of the source of the “special” character of phenomenal consciousness. Attention schema represents the attentional processes for the agent in a sparse manner, as its task is to control perceptual and cognitive systems, which does not require fully detailed representation. Dołęga and Dewhurst point out that this “frugality” is in fact a necessary requirement of a PP model of cognition—it ensures a proper balance between complexity and accuracy. According to the AST, this sparsity results in the “user illusion” (Dennett 1991) of there being something special, nonfunctional, and irreducible about phenomenal experience.

Against the WH account (discussed below), they further incorporate elements of Daniel Dennett’s multiple drafts view of consciousness (Dennett 1991), arguing that it is not simply the best hypothesis that becomes the contents of conscious perception. First, it is necessary for active inference to reduce the space of possibilities and then for attention to actually probe the hypotheses. This ties the account of “fame in the brain” (Dołęga and Dewhurst 2020) back to the AST, providing a mature deflationary theory of consciousness that treats the illusion of phenomenality seriously.

### The higher-order state space approach

The HOSS proposed by Stephen M. Fleming^11^ addresses directly the issue of reportability of the contents of subjective experience. Fleming proposes a computational model that accounts for the decision to report “I am aware of X” versus “I am unaware of X.” His central claim is that “awareness is a higher-order state in a generative model of perceptual contents” (Fleming 2020, 2). This means that awareness is explicitly included as a one-dimensional (discrete or continuous, if we allow for degrees of awareness) internal random variable in the generative model of a higher order than the variable tracking perceptual contents of experience. Simulations of Fleming’s remarkably simple model show its ability to account for phenomena of global ignition associated with awareness (an important element of different global workspace theories; see below).

Fleming’s model directly includes an asymmetry between being aware and unaware. The author claims that while being *aware* of an apple and of a hammer are two distinct states, both phenomenally and functionally, being *unaware* of apple and of hammer are very similar. This has consequences for prediction error in the PP account of perception: the hypothesis that the system is aware allows for much larger amount of prediction error since it invokes large belief updates within the generative model. Fleming’s simulations show these predictions to be correct and his interpretation takes the large Kullback–Leibler divergence, appearing when the system accepts the hypothesis “seen” versus “unseen” to correspond to the ignition pattern in the brain associated with reports of being aware.^12^

Fleming further suggests a connection to precision in a possible extension of his model. He points out that inference about the state of awareness can be aided by beliefs about attention and other states of the perceptual system, which he suggests can be implemented as beliefs about precision. In this way, he links his proposal with Graziano’s AST (see previous section), even though according to HOSS attention only provides “*input* into resolving ambiguity” (Fleming 2020, 7, italics original), and is not sufficient for determining awareness, which is a significant incongruency between the theories.

Fleming’s account, inasmuch as it can be expanded beyond the model of awareness it focuses on, builds on higher-order theories of consciousness (e.g. Lau 2007). This distinguishes this proposal from other Bayesian accounts of consciousness discussed here. HOSS takes consciousness to be a higher-order process of inference, which may take place without any active cognitive access to this information, e.g. in working memory. This provides limitations on the kind of cognitive architectures that allow for conscious processes to occur: they must allow for factorization along at least two lines (presence/absence and the contents of perception) of a rich multidimensional state space. This factorization may be computationally demanding and resultingly limit the kinds of cognitive systems that could be considered conscious. It also introduces complexity into HOSS, which can be taken to control how this factorization is performed by imposing an upper bound of the amount of states distinguished, although Fleming does not make this connection.

### The winning hypothesis account

The WH account of conscious perceptual experience has been originally hinted at in Jakob Hohwy and collaborators’ seminal paper on binocular rivalry (Hohwy *et al.* 2008; further developed in Hohwy 2012, 2013). It focuses on access consciousness (Block 1995) and does not discuss mental processes underlying phenomenal experience.

The main claim is that the contents of conscious experience are determined by the hypothesis about the state of the world, which has currently the highest posterior probability, i.e. the brain’s best guess. Each hypothesis about the state of the world can be assessed along two separate dimensions, with regard to first- and second-order statistics of the inference—namely, it can be both accurate and precise. Hohwy (2012) claims that, in order to constitute conscious perception, the hypothesis must score relatively high on both of them (see Fige 1 in Hohwy 2012), hence—since precisions in PP framework reflect attention, as described above—the percept must be actively attended by the subject.

This is best visible in the PP story of binocular rivalry, a perceptual phenomenon that occurs when subjects are presented with two different visual stimuli to each eye, e.g. a picture of a house and of a face. What subjects consciously perceive at any moment is either a face or a house and, over time, contents of perception fluctuate between the two images. PP (Hohwy *et al.* 2008) explains this phenomenon by indicating that both stimuli correspond to two separate hypotheses about the state of the world. Moreover, the hypotheses are mutually exclusive (due to strong prior expectations that no two objects can occupy the same space and that faces and houses are distinct objects). The brain must accept one of the guesses, leaving large amount of prediction error. This prediction error makes the inference unstable and finally results in a switch to a second best guess, which, however, does not account for all the inputs and remains unstable as well.

This account has been extended by Parr *et al.* (2019a) and applied to the phenomenon of Troxler fading, where static fixation leads to the experience of fading of peripheral stimuli. The choice of the WH is closely related to action and active inference (as discussed in more detail in the section on the GE account below). Parr and colleagues highlight the fact that binocular rivalry appears similar to Troxler fading, where perceptual switches are due to eyes shifting around the image—an overt action. In the former phenomenon, the shifts in percepts might be considered results of covert action, namely of attention shifting. This underscores the role of the control of precision in the WH account, creating a potential link not only to the GE approach but also to B-AST.

### Predictive Global Neuronal Workspace theory

The WH proposal has been further extended and integrated with the Global Neuronal Workspace theory [Dehaene and Changeux 2011; PGNW is developed by Hohwy (2013; 2015), Whyte (2019), and Whyte and Smith (2021)]. The two accounts are discussed separately, as the WH has also appeared throughout the literature as a distinct proposal, without reference to the larger apparatus of global workspace theory.

Global workspace is a proposition of a mechanism for information exchange between otherwise isolated brain regions and processes. The theory states that to become conscious, information must enter this global workspace implemented “in a network of densely connected pyramidal neurons possessing long-range excitatory axons connecting prefrontal and parietal cortices” (Whyte 2019, 4; see Dehaene and Changeux 2011). Serial information processing (resulting in experience of a “stream” of consciousness) comes from the inhibition of competing input processes. The central prediction of the model is that initial, local activity in the sensory cortices corresponds to unconscious stimulus processing, while conscious access appears in a relatively late time window relying on global activation in the prefronto-parietal network.

Dehaene (2008) proposed that the late ignition corresponds to the accumulation of evidence for a hypothesis about the state of the world. Hohwy (2013) further supplements this proposal with the role of action. In his account, ignition corresponds to the point at which a given hypothesis has accumulated sufficient amount of evidence to warrant a shift from perceptual to active inference (see also Whyte 2019). Selecting the best available policy of action requires that the hypothesis about the state of the world be held fixed. This indicates also that there should be top-down connections from the global workspace to sensory inputs, which carry hypotheses about the contents of consciousness. This approach offers a distinct hypothesis about the mechanism behind global ignition from the proposal of HOSS, and direct empirical comparison would be beneficial. One possible approach would be through designing an experiment with a fine control of granularity of state space of stimuli so that prediction of presence and absence would lead to equal amount of prediction error. In this case, according to HOSS, ignition would not in fact arise. However, given that the stimuli were chosen so that both their presence and absence elicits a demand for action—and active inference—if PGNW is on track, ignition would still arise, as if the brain fixed its best guess about the state of the world.

In an important study, Whyte and Smith (2021) provide simulations of visual consciousness with an implementation of the PGNW based on a recent version of the Active Inference Framework (Parr *et al.* 2019b). Their model simulates the electrophysiological and behavioral results from phenomenal masking and inattentional blindness experiments. This provides initial empirical support for the ideas behind the PGNW, as the results of simulations fit well with empirical data collected from human participants. In the paper, the authors also develop further the role of precisions in PGNW, showing that different levels of signal strength (precision of sensory likelihood a.k.a. sensory precision) and attention (precision of the hypothesis a.k.a. expected free energy precision) correspond to the standard taxonomy of factors influencing conscious access proposed within the GNW (Dehaene *et al.* 2006). Moreover, the authors expand the PGNW model by discussing the role of temporal depth, which is necessary in their account for the report of conscious experience (see also the Integrated World Modeling Theory section). In this way, they are able to provide a definition of conscious access in terms of an inferential process occurring at a sufficiently deep temporal level to allow for the integration and contextualization of information processing at less deep levels (Whyte and Smith 2021, 13).

Not only is the PGNW consistent with the neuroscientific evidence for the non-predictive version but it also provides several unique hypotheses that: (i) conscious representation should be continuous with processing at lower levels of the hierarchy, (ii) consistency of expectation influences the amplitude of P3 event-related potential component associated with subjective report (Whyte and Smith 2021, 12), and (iii) depending on the uncertainty about the stimuli, the dynamics of activity between prefrontal and parietal regions will be significantly different, corresponding to precisions assigned to either predictions (closer to the front of the brain) or prediction errors (in parietal regions) (Whyte 2019).

### The generative entanglement account

Andy Clark has developed a separate perspective on the problem of phenomenal consciousness (Clark 2018, 2019; Clark *et al.* 2019).

Clark’s account shares certain superficial features with the WH account in that the contents of consciousness depend on the cognitive systems’ “best hypothesis” about the state of the world. However, what sets this approach apart is the view of GE (Clark 2019) as the source of phenomenality. Given the task of predicting the state of the environment in which the subject—cognitive system itself—plays a significant causal role, there arises a need for the system to maintain a self-model able to predict its own states, “reactive complexes,” or “dispositions” (Clark 2019, 653) in response to certain structured patterns of sensory flows, in which interoceptive, exteroceptive, and proprioceptive inputs remain entangled. Because of this entanglement and resulting complexity, the reactive dispositions are modeled as coarse-grained, abbreviations or latent variables for those inflows. In other terms: they constitute the system’s mid-level hypothesis about the state of the world (Clark *et al.* 2019).

These “qualia” are puzzling to the agent for several reasons. First of all, these mid-level inferences underdetermine the actual state of the world while remaining highly certain to the system (i.e. they have high precisions assigned). In result, they are treated as mere “appearances” (Clark *et al.* 2019, 29), which nonetheless cannot be easily “shaken off.” Second of all, since these hypotheses model system’s own states, there is no need for a higher-level model explaining how these reactive dispositions came about—and in fact there is no such model (Clark 2019, 655). In result, the system has access only to the final estimation. “Qualia” are elements of a simplified self-model, which aims to be both accurate and concise in order to guide future choice and action.

Positing the contents of phenomenal consciousness on an intermediate or mid-level of the information processing hierarchy has a longer history, as it has been initially proposed by Jackendoff (1987) and later repeated by Prinz (2012). This view has often been criticized due to the difficulties with delineating the postulated intermediate level according to the criteria proposed (Marchi and Newen 2016). However, Marchi and Hohwy (2020) show that the notion of active inference provides clear-cut identification of the scope of phenomenal consciousness, which, in case of humans, happens to be in fact intermediate. This idea connects Clark’s GE back to Hohwy’s WH account. The contents of consciousness depend on the best hypothesis, which is held fixed for the purpose of active inference. At the same time, each organism has a privileged spatiotemporal scale on which the active inference operates, depending on the organization of the organism’s behavioral dispositions. In case of humans, Marchi and Hohwy identify this privileged scale with that of intuitively “basic” actions: reaching, grasping, taking a step, turning, crouching, etc. Hence, for active inference to be able to provide the organism with an optimal policy, the hypothesis specifying the contents of conscious experience has also to be spelled out on this spatiotemporal level. In case of humans, this in fact corresponds to the intermediate level, and the Marr’s “2.5d sketch” (Marr 1982; see Marchi and Hohwy 2020).

A slightly different proposal that fits with the scheme of the GE account has been earlier put forward by Jęczmińska (2017). Jęczmińska uses elements of the PP framework—in the version developed by Clark—to argue for a possibility of providing a novel, expanded theory of conscious experience through an intersection of Global Workspace Theory (in the version of Baars 1997) with the Sensorimotor Theory (as developed by O’Regan and Noë 2001). This account is, however, lacking details, and its main input boils down to underscoring the importance of the ongoing integration of data from different sensory modalities in the brain and its role in guiding action.

### Projective Consciousness Model

The PCM was originally conceived by David Rudrauf with several collaborators—most significantly Kenneth Williford and Daniel Bennequin (Rudrauf *et al.* 2017, 2020; Williford *et al.* 2018). While the model strongly relies on the view of mind advanced by the FEP, the central part of the explanation of consciousness it proposes comes from geometrical considerations.

The PCM accepts the view of mind advanced by the Active Inference Framework and its explanations of unconscious processing, control of behavior, and the functional role of consciousness—which is taken to be facilitation of FE minimization. However, the standard account of Active Inference Framework is expanded with discussions of the internal geometry of the generative model.

The PCM follows the program of (neuro)phenomenology in identifying phenomenal invariants of conscious experience (Williford *et al.* 2018, 3) and postulating them as axioms—as well as explananda—of the theory. In this way, the model accepts a conceptualization of the problem of consciousness focusing on the perspectival (in the spatial sense) and intentional elements of conscious experience. This perspectivality leads Rudrauf *et al.* (2017) to accept that the lived space is non-Euclidean and better described by projective geometry—a non-Euclidean geometry initially developed by Renaissance architects and painters and currently important in e.g. virtual reality and computer graphics research. Thus, according to the PCM, for the generative model to be capable to support conscious experience, it has to have the form prescribed by the projective geometry. More formally, they offer the concept of a “Field of Consciousness,” a three-dimensional projective space defined by a particular vector space corresponding to the point of view. The mental processes of perspective taking thus correspond to projective transformations (i.e. geometrical transformations of the space to a different point of view and its associated vector space), and the authors explicitly claim (Williford *et al.* 2018) that such transformations are computed by the brain (specifically by posterior cortical and subcortical structures; Rudrauf *et al.* 2017). The authors discuss several examples (e.g. visual illusions, such as Necker cube and out-of-body experiences) of application of the framework and support its plausibility with computer simulations.

This account declaratively connects the elements of global workspace theory as it assumes the central availability of conscious information with elements of the integrated information view (Rudrauf *et al.* 2017; Williford *et al.* 2018). Furthermore, it is compatible with some other accounts of consciousness within the Bayesian cognitive science, most specifically with the WH account, as Rudrauf *et al.* (2017) use the WH account to explain the uniqueness of the subject’s perspective (i.e. it is the one that is capable of explaining away the largest amount of the prediction error). The authors themselves identify “the thesis that projective transformations and projective frames necessarily subtend the appearance and workings of consciousness” (Williford *et al.* 2018, 15) as a key novel contribution of their model.

### Integrated World Modeling Theory

Adam Safron’s IWMT (Safron 2020) builds on the foundations provided by the Active Inference Framework and Integrated Information Theory (IIT) (e.g. Tononi *et al.* 2016), with—again—a strong influence of the Global Neuronal Workspace Theory (e.g. Dehaene 2014), as well as several others accounts of consciousness, including those discussed in this paper. Safron shows that IIT and Active Inference Framework are largely coherent and, specifically, that a union of the two accounts helps to solve some of the outstanding problems of each, such as e.g. the panpsychist consequences of integrated information view. The IWMT is a far-reaching, complex account of consciousness and, due to space considerations, I will have to omit some details.

To summarize this proposal, IWMT sees the essence of consciousness in a process “capable of generating a world model with spatial, temporal, and causal coherence with respect to the system and its causal inter-relations with its environment” (Safron 2020, 4). The postulated “integrative world models,” which sit at the core of this account connect postulates from Active Inference Framework, integrated information, and global neuronal workspace: they consist of generative models, with sufficient temporal and counterfactual depth, which are generated by complexes of integrated information and by global workspaces (understood as implementing Bayesian model selection). The integrative aspect refers to the spatial, temporal, causal, and agentive coherence, which are understood as ways of categorizing experience (giving the view a somewhat Kantian flavor).

On the algorithmic level, the key proposal of the IWMT are Self-Organizing Harmonic Modes (SOHMs), a mechanism for implementing complexes of integrated information, as well as global workspaces within the brain. The harmonic modes are synchronizations of activity of neural regions, providing effective connectivity between the said regions and potentially implementing self-evidencing [in the sense of Hohwy (2016)]. At the same time, according to the IWMT, they can explain both how particular perceptions cross the threshold of consciousness, as well as how unitary experiences emerge from probabilistic world models. Safron refers SOHMs to neural rhythms, with different functions—and experiential consequences—depending on the frequency bands.

Another mechanistic postulate of the theory is the implementation of PP in the cortex in the form of (folded) variational autoencoders^13^ in which the internal representation corresponds to inferred hidden causes. This somewhat standard account of neural implementation of PP is expanded through a reference to the turbo coding theory—a technical procedure of encoding signal to ensure maximal channel capacity through an introduction of redundant encoding (and decoding) of the signal by at least two encoders/decoders. The redundancy of information enables a feedback loop between the decoders, which provides the system with an error correction mechanism. In case of the cortex, turbo codes enable a connection between several circuits (modeled as folded autoencoders), providing them with a shared latent space that may be used e.g. for multimodal sensory integration. Although Safron does not discuss it directly, the reliance of IWMT on autoencoding—and the dimensionality reduction at the core of this algorithm—means that an essential feature of a generation of the world models is the reduction of the complexity of probability densities corresponding to them.

Phenomenologically speaking, IWMT ascribes to a form of a “non-Cartesian” theater, positing an internal, supposedly nonhomuncular, observer as a subject of the experiences. It envisages phenomenal experience as real, implemented by the state of the current maximal SOHM in the posteromedial cortices.

### Dual Aspect Monism

Solms (2019), building on earlier work with Friston (Solms and Friston 2018), offers yet a different take. According to the author, Chalmers’ hard problem of consciousness (Chalmers 1996) correctly points out that phenomenal consciousness cannot be reduced to function of vision or memory. However, for Solms, it does not mean that phenomenality itself is not functional or that it cannot be explained in functional terms.

His main point can be reconstructed as follows: both subjective experience of consciousness and physiological processes in the brain are different appearances of some other, abstract, functional process in the same way that lightning and thunder are different appearances of an electrical discharge. The case of children suffering from hydranencephaly, i.e. completely decorticated, in which case patients remain waking and their wakefulness has a certain qualitative—phenomenal—aspect, indicated by the fact that they are capable of affective states, leads Solms to identify consciousness with the function of “feeling” and locate it in subcortical structures that remain intact in the decorticated patients.

Solms argues that the function of feeling is to enable homeostatic control in unpredicted contexts, and states that “consciousness is *felt uncertainty*” (Solms 2019, 7). As this monitoring is related to survival, it is inherently value laden and classifies as good those actions and states that lead to survival (increase organism’s fitness). This leads both to subjectivity (i.e. availability only from the first-person perspective) as only the system itself can monitor its own internal states and to the qualitative nature of experience, which results from the compartmentalization of states of the organism into categorical variables, enabling the system to deal with the increasing complexity of its self-model. Finally, as conscious experience is influenced by contextual factors, precision weighting is necessary to control the prediction errors, and, hence, it is the mechanism implementing conscious access. Note, however, that, in Solms’ account, precisions are not identical with attention but rather implement both attention and motivation (Solms 2019, 12).

The crucial role played by the homeostatic sources of experience in Solms’ model makes an interesting connection to a previous attempt to connect PP framework to the Global Neuronal Workspace theory; the proposal of the “limbic workspace” (Chanes and Barrett 2016; as Whyte 2019 points out this proposal differs from PGNW discussed above with regard to postulated model and its predictions). Chanes and Barrett (2016) focus on the role that limbic cortices (e.g. anterior insula, parahippocampal gyrus, and cingulate cortex) play in cortical processing and conscious access. The authors suggest that limbic cortices select between representations, factoring in the organism’s homeostatic needs and preferences. Moreover, since these brain areas have bidirectional connections to subcortical structures, as well as other cortical regions, they seem to be a plausible extension of Solms’ model, connecting his explanation of “feeling” to the processing in brain cortex, showing how mental images could be imbued with a “what it’s like.” If such a connection would turn out to be plausible, it would significantly expand the scope of Solms’ original account.

### Markovian Monism

MM (Friston *et al.* 2020) is advanced from the first principles (Colombo and Wright 2018) as a theory of the origin of consciousness. It does not provide any mechanistic nor implementational details for the actual realization of consciousness, but rather it shows how sentience (in a weak sense of responding to sensory impressions) is entailed by the tenets of the FEP. Specifically, it claims that sentience follows from the existence of the Markov blanket. Furthermore, it is an explicitly metaphysical account of phenomenality (but see the criticism of Beni 2021). While it differs in scope and approach from other papers discussed here, it provides several important insights into the general picture of consciousness sketched by the Bayesian cognitive science and, as such, requires some discussion.

In this review, I will focus on two features of this account: the Cartesian duality of mind with its description in terms of information geometry and Friston and collaborators’ reference to the central role of precision for consciousness.

The Cartesian duality of the mind can be cast in terms of a possibility of describing the cognitive system from two perspectives: a third-person perspective of psychology, neuroscience, etc., as well as from the first-person perspective of subjective experience. According to the FEP, this division appears when we consider any system as delineated by a Markov blanket—i.e. the set of states or variables that render the internal states of the system conditionally independent from anything else. While Friston argues [e.g. in his monograph (Friston 2019); but see Bruineberg *et al.* (2020) for critical analysis of this proposal] that the existence of the Markov blanket is necessary for the existence of every “thing” as far as it can be distinguished from everything else, a key insight here comes from the fact that the delineated system can be described in terms of gradient flow on self-information or surprisal (roughly speaking, this is possible as it is the steady-state solution to the Fokker–Planck equation, a standard description of time evolution of dynamical systems). Together with the very existence of the Markov blanket, this provides the modeler with the possibility of describing the internal states as modeling external states, precisely in the manner described in the introduction to the FEP above. If we equip these statistical manifolds with a Fisher information metric (which quantifies the distance between two points by accumulating the Kullback–Leibler divergence between distributions encoded on the manifold *en route* from one point to the other), we impose an *information geometry* onto the Markov blanket delineated system; but as we have two independent densities, we, in fact, need to define two information geometries—*intrinsic*, describing autonomous (i.e. internal and active) states, parameterized by time, and *extrinsic*, describing external states and parameterized by beliefs—i.e. internal states.

While this is only an account of why conscious experience appears in principle, omitting its structure and implementation in the case of actual human experience, Friston *et al.* (2020, 21–22) argue that not only does it show the origin of sentience and account for the private, subjective character of phenomenal qualities but it also entails several of the criteria of consciousness, as characterized by the IIT (e.g. uniqueness and unity of conscious experience).

More specific details of neural implementation of consciousness are only briefly discussed. The authors point out that the Fisher information metric on the internal statistical manifold is the curvature of the variational free energy. Furthermore, they claim that this is the same parameter as the precision or confidence of beliefs about external states. This parallel is not worked out in formal detail but rather attributed to the work on gauge theories (Sengupta *et al.* 2016); however, even the informal definition of Fisher information metric provided above should give the reader the general idea of the considerations showing this equivalence. This makes a direct reference between the work on MM and the work on DAM described above, as well as several other papers claiming e.g. that precision plays a key role in opacity or transparency of phenomenal beliefs (Limanowski and Friston 2018).

## Discussion

Bayesian cognitive science offers a hodgepodge of approaches to the study of consciousness, which differ in the definition of the problem, the extent of phenomena covered, the level of formal and/or implementational detail, as well as in metaphysical commitments of the views. These differences are briefly summarized in Table 1. This picture fits well with the “orthogonality” of PP approaches with regard to current debates in neuroscience and philosophy of mind, as stated by Jakob Hohwy (Hohwy 2020, 11). Furthermore, this duality is actively exploited by researchers, who not only attempt to connect PP accounts with extant prevailing theories of consciousness, such as global neuronal workspace theory, or IIT,^14^ but also believe that FEP can build bridges between those, somewhat inconsistent, accounts.

**Table 1.** The table summarizes the scope of the theories discussed in this review

Theory	Access	Phenomenal	Function	Origins	Algorithm	Implementation
B-AST	X	X	X		?	?
HOSS	?				X	?
WH	X				X	
PGNW	X		X		X	X
GE	X	X	X	?		
PCM	X	X	X		X	X
IWMT	X	X	X	?	X	X
DAM	X	X	X			X
MM				X		

“X” marks whether the theory provides (or at least attempts to provide) an account of: access consciousness, phenomenal consciousness, function of consciousness, origins of consciousness (understood in terms of a possible “evolutionary trajectory” of forms of sentience of increasing complexity), the algorithmic description of postulated processes, and a description of possible implementation of this algorithm (roughly in the standard sense of Marr 1982). In problematic cases, when one of those explananda is not directly engaged with but rather hinted at, question mark was used.

I will now turn to the discussion of the “larger picture” that the Bayesian cognitive scientists working on consciousness paint, some problems it encounters, and its possible interpretation in terms of a minimal unifying model.

### Bayesian cognitive science of consciousness

What becomes clear once we discuss all of these accounts together is that, despite their differences, their formal solutions to the specific phenomena of consciousness are more or less the same between the accounts. Specifically, they all agree that precision (or precision optimization) and complexity of the internal model are responsible for access and phenomenal consciousness. The general picture is the following:

1. The contents of conscious perception depend upon the posterior probability assigned to different hypotheses about the state of the world (B-AST, WH, PGNW, GE, and PCM) and
2. the landscape of possibilities is reduced to serial processing due to the exigencies of action (WH and GE),
  - however, access to this selected hypothesis is managed by precision weighting of different processing streams, which can take the form of attention (B-AST, HOSS, and PGNW) and possibly happens *post* *hoc*, i.e.—at the point of storage (B-AST and DAM).
3. In order to efficiently manage precisions, a higher-order model for precision optimization is required. It can take the form of a meta-model (WH), a self-model (GE and DAM), an attention schema (B-AST), or a higher-order state in the model (HOSS).
4. The contents of this model are maintained on an intermediate level, which balances out precision and complexity and allows active inference to provide optimal policies (B-AST, GE, IWMT, and DAM). At the same time, the model is not accessed by any kind of higher level process and, as such, remains at the top level of information processing hierarchy (B-AST, HOSS, GE, and IWMT) even if its contents are passed on for further processing.
5. The structure of this model is the source of the “special,” “ineffable” character of phenomenal experience (B-AST, GE, and IWMT),
  - however, it does not necessarily diffuse the metaphysical reality of the predictions of this model, taking the form of qualia (GE) or feelings (DAM), which are as real as perceptions of objects such as “cats” or “computers” (GE).

There are obviously points of important disagreement. To note only the most extensive:

The PCM does not assign the existence of phenomenal consciousness to the meta-model of precision but rather to the generative density itself (it dissents with regard to points 3–5). The “special,” “ineffable,” and perspectival character of experience comes from the geometrical structure of the model. Note, however, that it is formally possible that one of the effects of the geometrical transformations postulated by PCM is a reduction of complexity of the model as these transformations focus on what could be called the “syntactic” structure of perception while eschewing some level of detail. A possible argument for this interpretation would focus on the fact that the projective transformations postulated by the PCM do not necessarily preserve the identity of transformed objects. Furthermore, to support this idea, one could argue that the transformation postulated by this model is not directly computed in the brain, but rather approximated, using a representation with reduced complexity. Following this argument is, however, outside the scope of this paper, and furthermore—this dissension of projective consciousness has its (conceptual) advantages for the Bayesian theories of consciousness at large.

The IWMT does not explicitly connect access consciousness to attention and precision, positing the self-organizing harmonic modes as the key mechanism (it dissents with regard to points 1–2). While Safron’s considerations—especially the hypotheses about the role of specific frequencies of neural rhythms—enable us to directly connect self-organizing harmonic modes with attentional processing (mainly because of the relationship between the harmonic modes and alpha and beta EEG frequency bands, which are associated with attentional processing in the literature; see e.g. Klimesch 2012), the relationship between these “metastable synchronous complexes of effective connectivity” and precision optimization is more convoluted. Functionally, it seems plausible to regard the self-organizing harmonic modes as akin to an attention schema as they “may act as systemic causes in selecting specific dynamics through synchronous signal amplification” (Safron 2020, 14). However, since, formally, they are defined in terms of attractors for phase space description of neural activity, it is difficult to see how they could be deconstructed into specific variables of the said neural system, including precision, due to the interdependencies of variables in the system. Hence, precision is not usually explicitly modeled by the self-organizing harmonic modes, making the IWMT distinct from the other FEP approaches to attention. Instead, precision optimization is performed in a distributed fashion, as a by-product of multiple neural processes: both an “as if,” implicit management of attention, and explicit, conscious and unconscious, direct “attentional shifts.” What follows is that access to consciousness is not directly implemented by attentional biasing but rather by a more generic process. This also bears some similarities to the HOSS, where Fleming (2020) underscores that precision optimization is not the only mechanism responsible for awareness. This view is in fact more in line with the current state-of-the-art research on the role of attention for consciousness, which underscores the importance of differentiating between different types of attentional processing (see e.g. Pitts *et al.* 2018).^15^

The MM is the great absent of the list above, as it remains silent on these issues, focusing on providing formal foundations for the more detailed models.

There are of course other similarities shared less widely by only two or three of the proposals discussed here. Their limited scope does not warrant inclusion in the general picture painted above; however, it is important to note some of the emerging proponents like temporal depth (related to complexity), whose importance has been underscored e.g. in recent developments of the PGNW (Whyte and Smith 2021) and in IWMT.

Finally, it is important to note that if one were to restrict to only a subset of approaches within Bayesian cognitive science and to concepts exclusive to the framework, the general picture could differ significantly. In a recent article, Vilas *et al.* (2021) review explanations of various phenomena of consciousness specifically from the Active Inference Framework and develop a significantly different picture from the one proposed above, one relying heavily on the structure of the generative model, in particular its temporal and counterfactual depth. However, as the authors accurately point out, once we limit ourselves to Active Inference Framework only, the emerging picture remains preliminary and needs to be extended with mechanistic and implementational details.

A cross-section of the accounts discussed in this review provides us with a general view of consciousness that all Bayesian cognitive science theories are likely to be congruent with. However, I believe that a more profound reading of these similarities is justified. Namely, the postulated role of precision and complexity can be regarded as Minimal Unifying Model of consciousness (MUM; in the sense of Wiese 2020).

### Can Bayesian cognitive science alone provide a theory of consciousness?

Before turning to the discussion of the MUM, one important objection needs to be noted.^16^ In a recent paper, Marvan and Havlík (2021) analyze some of the accounts of consciousness discussed here. Their goal is to argue that PP does not by itself provide a theory of consciousness but requires external explanatory machinery provided by established theories of consciousness to do so. Given the detailed overview of accounts of consciousness within Bayesian cognitive science provided here, their criticism seems to be to some degree correct. Out of those theories, perhaps, only the WH, the GE accounts, DAM, and MM do not make explicit references to some theory of consciousness “external” to the framework, although the last account is outside of the scope of Marvan and Havlík interests (for reasons already discussed). Marvan and Havlík directly criticize the main claim of the WH approach, pointing toward incompatible evidence from blindsight (see also Dołęga and Dewhurst 2020 criticism) and continuous flash suppression paradigm (Tsuchiya and Koch 2005). In this way, they reject also the GE account. DAM, however, is not mentioned by them and seems immune to their objections. In this case, conscious access is implemented by only precision weighing, which is a “proprietary” idea of Bayesian cognitive science and could provide an explanation of continuous flash suppression. In very informal terms, one could point out that continuous flashing even to one eye reduces the precision assigned to all information coming from visual modality. This happens since the brain starts to classify these (unlikely) sensory inputs as noisy and unreliable and, normally, there is a correspondence between the reliability of both eyes, leading to distrusting both eyes. In result, it discards the correct hypothesis explaining the pattern presented to the second eye. This kind of explanation could be experimentally tested with participants who have worse eyesight in one eye—in this case, flashing should cause significantly weaker suppression as the brain most likely would model the precision of each eye separately. Alternatively, the absence of suppression in a multimodal version of continuous flash suppression could provide support for this hypothesis.

Furthermore, some of the accounts that refer to other theories of consciousness make connections to “external” accounts without depending on those theories’ explanatory tools, as in the case of e.g. Fleming’s HOSS and the recent take on the PGNW due to Whyte and Smith (2021). In these two cases, in fact, PP is capable of providing an explanation of the source of ignition associated with awareness rather than rely on this notion to explain conscious access. In HOSS, as discussed previously, ignition is taken to be associated with inference and asymmetrical prediction error appearing when the higher-order random variable tracking awareness accepts hypothesis “seen.” In PGNW, the phenomenon of ignition is reproduced by the model when it is presented with the stimulus. Whyte and Smith connect ignition in their model with evidence accumulation and belief update at temporally deep levels of their generative model. In this way, Bayesian cognitive science of consciousness escapes Marvan and Havlík’s criticism but furthermore is able to supplement more established theories with mechanistic details.

Finally, while it is common to treat PP as a *theory* of cognition, with strong unifying ambitions, such a perspective is in fact very controversial. The unificatory ambitions have been pointed out as failed (Litwin and Miłkowski 2020) and, among the recent shift toward more instrumental understanding of the FEP (Colombo and Wright 2018; van Es 2020; Andrews 2021), it has been also suggested that PP does not hold as a “theory” in the first place but should rather be considered a framework or a toolbox (Litwin and Miłkowski 2020). While this point is debatable in relation to PP on its own, it is much less so in the context of Active Inference Framework, the FEP, and the Bayesian cognitive science at large. If we take such a perspective, Marvan and Havlík objections are correct, yet misguided, since there was never a need for Bayesian cognitive science to provide theory of consciousness all by itself. This perspective seems also the most charitable for the interpretation of precision and complexity in terms of a minimal unifying model, as I will argue below.

## Precision and complexity as a minimal unifying model

Wiese (2020) proposes the notion of the minimal unifying model as “a model that

1. specifies only necessary properties of consciousness (i.e. it does not entail a strong sufficiency claim),
2. has determinable descriptions that can be made more specific, and
3. integrates existing approaches to consciousness by highlighting common assumptions” (Wiese 2020, 2).

I argue that precision and complexity can be seen as elements of exactly such a model, complementary to the information generation account that Wiese discusses. The relation between Wiese’s account and the one proposed here is the following: since Wiese posits only that MUM should specify *necessary* properties of consciousness, information generation (Kanai *et al.* 2019) can be easily supplemented by the notions of precision and complexity in the manner discussed below (see the end of the “Conditions for a MUM” section). Furthermore, these two proposals are logically independent to a large degree. Should empirical evidence lead us to reject either information generation or precision and complexity as unnecessary for conscious experience, the other element can still be considered a MUM.

To flesh out my proposal, I will show that the roles that precision (optimization) and complexity (limiting) play for, respectively, access and phenomenal consciousness do indeed meet the three conditions set by Wiese. In doing so, I am abstracting away from the details of particular views discussed earlier in the paper and focusing on the core of the Bayesian approach to consciousness outlined in the “Bayesian cognitive science of consciousness” section. The key point is to show that these two features are in fact necessary for consciousness under this general Bayesian approach. Then, I will analyze whether precision and complexity can be considered a MUM independently of the Bayesian cognitive science that they were developed as a part of similar to the way that Wiese disjoints information generation from the larger proposal of Kanai and colleagues.

### Conditions for a MUM

The first condition states that MUM should consist of necessary but not sufficient features. Here, I will argue briefly against the sufficiency of precision and complexity and turn to its necessary character in the next section as it is closely related to the connection between Bayesian cognitive science and the current proposal.

While all accounts discussed above assign a strong explanatory role to precision and complexity, each of them provides further specifications and limitations with regard to relevant properties. This in fact has been the subject of the criticisms put forward by Marvan and Havlík (2021). The WH account is closest to establishing precision optimization as sufficient for conscious access. However, the simulations by Parr *et al.* (2019a), as well as the work of Marchi and Hohwy (2020), point out that while precision is responsible for managing current contents of conscious experience, action and active inference are further required to specify any particular percept. Most strongly, the insufficient character of precision is underscored by Fleming in his HOSS, where he explicitly points out that precision provides input into resolving ambiguity but does not determine awareness. Complexity, on the other hand, is strongly entangled with the processing of hypotheses and updating of the generative model and, while it can be conceptually disconnected (as shown below), it only plays a role of a limit or a reference point for other processes and, hence, cannot be considered a sufficient condition of consciousness.

The second condition is met quite obviously as both “precision” and “complexity” are well-defined terms within the formalism of Bayesian cognitive science. There are multiple competing models arguing for specific neural or at least algorithmic interpretations of these processes (see e.g. Spratling 2017). Regarded independently of this framework, they still retain their strong, mathematical definitions and can be fitted into multiple different theories of consciousness as exhibited by the different connections Bayesian cognitive science makes to established accounts of consciousness.

Finally, the third condition states that MUM should allow for making connections between existing accounts of consciousness. We can consider this condition on at least two levels.

First of all, if we accept the broad assumption that something sufficiently similar to the Bayesian cognitive science is a correct theory of cognition, several of the discussed accounts make claims (with different degree of evidence cited to support them) that the FEP can be employed to build bridges between predominant theories of consciousness. It should suffice to refer to B-AST, PGNW, and IWMT. Furthermore, the connections to theories such as global neuronal workspace or IIT build upon precisely the notions of precision and complexity.

Second of all, as mentioned above, understanding precision and complexity as a MUM is complementary, rather than competitive, to the information generation theory of Kanai *et al.* (2019).^17^ The Kanai *et al.* (2019) view can be summarized as a view that the basic function of consciousness consists of generating representations of events that are possibly spatiotemporally detached. Information generation in this model follows from a compression–decompression view of perception, algorithmically described by variational autoencoders. To describe what kind of information it is, the authors employ the notion of a generative model, in the sense of FEP described above, and Wiese makes a connection to the algorithmic information theory of consciousness (or simply Kolmogorov theory), advanced by Ruffini (2017), which helps clarify the compression–decompression view, and introduces the notion of complexity into the MUM advanced by Wiese.

It is important to note that this is a different notion of complexity and we should be wary of conflating it with the one employed in Bayesian cognitive science. Ruffini (2017) and Wiese (2020) use the term “complexity” in the technical sense of Kolmogorov complexity (see Cover and Thomas 2006). The two notions are closely related as they both provide quantifications of the amount of information loss (a formal treatment of those similarities is provided by e.g. Cerra and Datcu 2011). Furthermore, they are both (in general) incomputable and can be approximated in terms of data compression. The most important difference is that the notion of “relative entropy” (Kullback–Leibler divergence seen in the free energy equation) is specified within the probabilistic framework of classical information theory and shares its inability to “describe information content of an isolated object” (Cerra and Datcu 2011, 903). Kolmogorov complexity, on the other hand, is defined within algorithmic information theory, which takes the notion of information content of an isolated object as a primary concept (Cerra and Datcu 2011, 903). Cerra and Datcu show that it is possible to construct a parallel for the notion of relative entropy within the algorithmic information theory, defined on the basis of Kolmogorov complexity, as “the compression power which is lost when using such a representation for [a string] x instead of its most compact one, which has length equal to its Kolmogorov complexity” (903).

Despite these different backgrounds, both notions of complexity play similar roles. For Ruffini (2017), “structured conscious awareness is associated to information processing systems that are efficient in describing and interacting with the external world (information)” (5), where efficiency is described as low (Kolmogorov) complexity of incoming data given the internal model. In Bayesian cognitive science, complexity similarly is used to account for “efficiency” of the generative model. Hence, despite different formal definitions employed, considering complexity as a MUM is compatible with information generation theory. Furthermore, the inclusion of precision optimization to this account would be straightforward, as shown below.

### Precision and complexity (optimization) as necessary properties of consciousness

The question of whether precision and complexity optimization can be considered *necessary* properties of a conscious system depends to a degree on our acceptance of the Bayesian cognitive science story of cognition. If one agrees that this theory provides a sufficiently adequate model of cognitive systems, then the answer to this question is obvious, as these are central terms in Bayesian cognitive science and even more so in its account of conscious experience. However, it is not the case once we consider a broader picture. While Wiese (2020) argues that all theories of consciousness have to contain the component of information generation, and this claim seems uncontroversial, the same cannot be said about precision and complexity. For example, it is an empirical question whether a theory that would posit that the complexity of an internal model [in a very general sense, akin to what is required by Kanai *et al.* (2019) and Ruffini (2017)] is set to a constant value or that the cognitive system is incapable of estimating and managing precision of incoming inputs would be capable of explaining consciousness (the latter might be the case in IWMT, as mentioned above). However, at the same time, the postulates of precision and complexity optimization are not limited to an acceptance of a full Bayesian cognitive science story about the mind and they can supplement other accounts. It is especially so if we accept that these ideas form a framework rather than a unifying theory of mind (see Litwin and Miłkowski 2020).

Consider again the information generation theory (Kanai *et al.* 2019) and precision optimization. In constructing their theory, Kanai and colleagues specifically aim to address the aspect of consciousness concerned with the broadcast of information [as opposed to metacognition, distinction put forward by Dehaene *et al.* (2017)]. They argue that what underlies functions associated with global broadcast is the “capability of generating fictional representations using internal, sensorimotor models” (Kanai *et al.* 2019, 3). What their account leaves out and one more reason to conclude that it presents necessary but not sufficient condition for conscious experience is precisely the question of how this generated information becomes available across the brain. Kanai and collaborators simply state that this information is maintained in short-term memory, placing the central question of the global workspace theory outside of the scope of their interests. However, since information generation can be implemented in a predictive coding scheme, it is straightforward to employ the tools of PP to explain the short-term memory in terms of precisions (or weights) assigned to perceived versus generated information.

We have seen, similarly, how precision and complexity can be related to the global neuronal workspace theory and the IIT. Hence, the claim of this paper can be read in two ways.

First, in a relatively uncontroversial but limited way:

Precision and complexity constitute a MUM for Bayesian cognitive science of consciousness.

In the manner described by Wiese, casting common elements of the Bayesian theories of consciousness in terms of a minimal unifying model helps unify them [in the sense of Miłkowski (2016) and Miłkowski and Hohol (2020)] with existing theories of consciousness—in this case, also with formerly proposed minimal unifying model. In this way, precision and complexity optimization become key elements for future experiments seeking to test Bayesian theories of consciousness. In the manner already spearheaded in the studies of Whyte and Smith (2021) and Parr *et al.* (2019a), such experiments should seek to reproduce results of behavioral and imaging studies within standard paradigms employed in the study of consciousness. Initial experiments should focus especially on the role of precision and complexity to validate the widely Bayesian approach. This could be done by e.g. repeating the studies of Fleming (2020) with explicit modeling of precision estimation, instead of assuming fixed noise (as Fleming does).

Second, in a broader but fairly controversial way:

Precision and complexity constitute a MUM of consciousness in general.

The close relation between Bayesian approaches to consciousness and the more classical accounts suggests that once we accept the interpretation of Bayesian cognitive science in terms of a framework or a toolbox, there is a broad group of theories that may be extended with precision and complexity. In fact, precision assignments can be included in almost any information processing-based account of consciousness, which includes variance, entropy, or another term playing a comparable role—i.e. almost any existing account. Complexity posits additional restrictions as it requires the account to postulate an internal model but only in a very broad sense—again, met by a plethora of existing accounts. This interpretation, however, might be taken to weaken the technical sense of these two terms and in result—to restrict the amount of the explanatory work they can do. Furthermore, adding precision and complexity to existing theories can have a *post hoc*, reinterpretative, and redundant character. However, as has been shown throughout this paper, there are some instances where these two concepts can contribute to deliver novel, testable explanations of phenomena and properties of consciousness for more classical accounts. One such plausible example comes from the explanations of global ignition provided by HOSS and PGNW. Both suggest a plausible mechanism underlying ignition, often glossed over within global neuronal workspace theory, and both could be empirically validated outside of the setting of Bayesian cognitive science in the manner described in a previous section. If this research program were to succeed, it would further secure a strong foothold for the Bayesian cognitive science as a useful toolbox for explaining mechanisms underlying cognition.

## Data Availability

Data availability is not applicable.

## Footnotes

1 The distinction between phenomenal and access consciousness is well established but widely disputed (e.g. Kriegel 2007; Overgaard 2018); hence, I use it throughout the paper only for the purpose of framing the discussion.

2 While it is important to note both the different epistemic status of FEP and PP and Active Inference Framework and some philosophical as well as mathematical discrepancies between these approaches, for the purpose of current discussion, these differences may be safely disregarded as I will be addressing their very theoretical core. Some of the more important differences will be marked throughout the text as necessary. An important point to note here is that, while the notion of “active inference” is applied variously in the literature to describe either the process of active inference (discussed in the next section), a theory of motor control (e.g. Adams *et al.* 2013), as well as a framework for modeling decision-making, perception, and action selection with partially observable Markov decision processes, in continuous, discrete, and mixed-state spaces, with somewhat similar goals to the reinforcement learning approach in machine learning [see e.g. Sajid *et al.* (2021) for a review of applications in discrete cases and Friston *et al.* (2017b) for a discussion of the continuous and mixed cases]. In this paper, I use “active inference” to denote the first meaning and “Active Inference Framework” to denote the last. I am grateful to an anonymous reviewer for pressing me to clarify this.

3 Depending on the accepted stance in the internalist/externalist debates, various authors differently describe the generative model as being employed or encoded by the system [in the case of the neurocentric version of PP, e.g. see Kiefer and Hohwy (2018)] or embodied or instantiated by the system (in the case of the embodied and enactive version of PP and in the case of FEP at large, e.g. see Ramstead *et al.* 2020). For the discussion of this distinction, see van Es (2020).

4 Formal parts of this review are based on Buckley *et al.* (2017). Furthermore, it is important to note that the FEP is undergoing a continuous development and improvement and, since this brief introduction is aimed at introducing key elements of the formalism, there might be some discrepancies between what is presented here and formal parts of the works reviewed below, which have, in some cases, been done at a different stage of the framework. This introduction attempts to capture the most recent version of FEP, making use of the “generalized free energy” term while at the same time ignoring some of the (important) differences between FEP, PP, and Active Inference Framework (see e.g. Ramstead *et al.* 2020) as already mentioned.

5 The “great absent” of this discussion is the notion of a *Markov blanket* that is crucial for the contemporary version of FEP. However, most of the accounts discussed here do not make any reference to this concept; hence, I have decided to omit it to keep the discussion simple. It is briefly introduced in the section discussing MM below, and an interested reader is directed to papers that focus on developing this concept, e.g. Kirchhoff *et al.* (2018) and Parr *et al.* (2020), and to the critical discussion in Bruineberg *et al.* (2020).

6 This applies to the standard approach of Bayesian cognitive science, which employs continuous probability densities, which is the main focus of this paper. In different, equivalent formulations, e.g. based on categorical distributions (see Kwisthout and van Rooij 2015; Friston *et al.* 2015b; Kwisthout *et al.* 2017), the formal definition of precision can differ, although the role it plays in the formalism remains comparable. For example, when categorical distributions are involved, precision is described in terms of entropy.

7 I am grateful to an anonymous reviewer for pressing this issue.

8 Smith *et al.* (2021) replace the term “precision over policies,” proposed and used by Parr and Friston, with “expected free energy precision” to highlight the fact that this factor modulates the contribution of the expected free energy term to the posterior distribution over policies. This further allows to avoid confusion with terminology used in reinforcement learning. For this reason, in this paper, I will follow Smith and colleagues’ terminology in this regard.

This is an attempt at an exhaustive list of accounts of consciousness within the Bayesian cognitive science. It is based on the works collected by Hohwy (2020, Supplemental Table S1) with some newer publications added. As the field is dynamically developing, I apologize in advance for omitting any proposal. It is important to mark here that I am well aware of a strong bias with regard to the authors of the views discussed here. I have taken several steps to avoid it (thorough research and crowdsourcing the list of publications to be discussed), which suggests that the bias is not an artifact of this review but rather—unfortunately—exists in the field.

10 It could be objected here that the flexibility of Bayesian cognitive science is in fact problematic and that postulated models of consciousness boil down to a simple redescription of existing accounts with *post hoc* adjustments. This objection refers to broader arguments brought up against the Bayesian cognitive science: the objection of the tautological nature of the FEP (but see Andrews 2021) and of the *ad hoc* and redescriptive nature of at least some of the explanations within this framework (raised by Litwin and Miłkowski 2020; Cao 2020). In case of consciousness, however, this is not true. Even the theories that are closest to be seen as mere redescriptions (e.g. PGNW and B-AST) offer novel predictions and algorithms, which enable to distinguish them from their non-Bayesian “ancestors.” At the same time, most of the theories puts forward novel theoretical ideas, even if they (in fact quite often) stem from combining approaches hitherto considered opposed. Hence, this objection does not seem to be a serious argument against the approaches discussed here.

11 I am grateful to anonymous reviewers for pointing me towards Fleming’s paper.

12 An objection should be noted, however. Fleming’s interpretation of large error as corresponding to ignition hinges on the implementation of PP algorithms within the brain, the issue which Fleming does not elaborate upon. Standard views on the implementation of the PP algorithms in the brain assume the existence of specialized error neurons that encode prediction error (see e.g. Bastos *et al.* 2012; Spratling 2017, 2019). If that is the case, a large amount of prediction error provides only an indirect explanation of global ignition, since it implies only activity of this one type of neuron, which need not lead to global ignition of the type Fleming envisages. Nonetheless, Fleming’s model deserves attention, as it provides a very elegant explanation of a widely studied phenomenon.

13 Autoencoder (see e.g. Goodfellow *et al.* 2016, 493–516) is a type of an artificial neural network that is trained in an unsupervised fashion to learn to copy its input into its output through an internal representation of different (usually lower) dimensionality than the input. In this way, autoencoders are capable of learning efficient coding of inputs. Variational autoencoders employ generative models to this goal. “Folded” in this context describes the topology of the artificial neural network implementing the autoencoder, which is folded at the internal representation so that the neurons implementing encoding and decoding parts of the processing are aligned (see figure 2 in Safron 2020).

14 While IIT has been less represented among the views discussed here than other competing theories, a recent preprint (Waade *et al.* 2020) discusses another Bayesian approach to consciousness currently under development, which is likely to strengthen the position of IIT in this context.

15 I am greatly indebted to Adam Safron for the discussion of some of the finer details of the IWMT related to the relationship between self-organizing harmonic modes, attention, and access consciousness.

16 I am grateful to an anonymous reviewer for pointing me towards this paper and suggesting that HOSS and PGNW are somewhat immune to those objections.

17 I do not count Kanai *et al.*’s (2019) proposal as falling within the Bayesian cognitive science as it makes reference to the predictive coding schema only in terms of implementation of postulated mechanisms.
